# Species diversity of coral reef fishes around the West Island of Sanya City, South China Sea, based on environmental DNA

**DOI:** 10.3897/BDJ.10.e89685

**Published:** 2022-09-30

**Authors:** Rui Xi, Wanru Gao, Xin Wang, Yingchun Xing

**Affiliations:** 1 National Demonstration Center for Experimental Fisheries Science Education, Shanghai Ocean University, Shanghai 201306, China National Demonstration Center for Experimental Fisheries Science Education, Shanghai Ocean University Shanghai 201306 China; 2 Resource and Environmental Research Center, Chinese Academy of Fishery Sciences, Beijing 100141, China Resource and Environmental Research Center, Chinese Academy of Fishery Sciences Beijing 100141 China; 3 Hainan Fisheries Innovation Research Institute, Chinese Academy of Fishery Sciences, Sanya 572024, China Hainan Fisheries Innovation Research Institute, Chinese Academy of Fishery Sciences Sanya 572024 China

**Keywords:** coral reef ecosystem, occurrence of fish taxon, distribution, habitat condition, sequences of fish MOTUs, South China Sea

## Abstract

**Background:**

West Island is the second largest island in the Hainan Province, China and its surrounding sea area has a well-preserved coral reef ecosystem and high species diversity of coral reef fishes. Undoubtedly, coral reef fishes and coral reefs have complex symbiotic relationships and fish species diversity should reflect the healthy status of coral reef ecosystems. Environmental DNA (eDNA) is a useful and sensitive tool to detect fish species and causes less environmental damage than traditional fish survey methods. This paper investigated coral reef fish species of West Island, Hainan Province, China, based on eDNA and provided scientific data for understanding and protection of the coral reef ecosystem of the South China Sea.

**New information:**

The sea area surrounding West Island is the most important component of the coral reef ecosystem in the northern part of the South China Sea, which is also an essential part of the largest National Coral Reef Reserve in China. However, complete data of fish species distributed in this region have been a long-term gap. This study provides information on 41 fish species belonging to 28 genera, 16 families and three orders in this sea area and is the first complete record of coral reef fishes surrounding the West Island. In addition, the information of Molecular Operational Taxonomic Units (MOTUs) for taxon identification were also provided and it could contribute to building specific eDNA taxonomy database of coral reef fishes of the South China Sea. The study includes three datasets, with aspects of fish taxon-occurrences, MOTUs sequences and information of environmental indicators surrounding West Island, Hainan Province, China. The “fish taxon occurrences” dataset presents records involving taxonomic, distribution, habitat condition, latitude and longitude of 41 coral reef fish species detected, based on eDNA, the “MOTUs sequences” dataset provides MOTUs sequences and their abundance of 31 species detected and the “information of environmental indicators” dataset presents records of transparency, temperature, water pressure, dissolved oxygen, electrical conductivity, hydrogen and redox potential measured from five sampling localities.

## Introduction

The coral reef is an important tropical marine ecosystem and it provides abundant habitats for fish communities (Choi et al. 2020). Hainan Island (Spalding et al. 2007) is located at the South China Sea, harbouring nearly 2 million square kilometres of sea area and provides suitable climatic, physiological and biochemical conditions for the development of large-scale coral reefs in China (Roder et al. 2012, Lin et al. 2022).The coral reef fishes are a most crucial component of coral reef ecosystems and they play a key role in the maintaining ecological function (Cook et al. 2022). Therefore, research on their species diversity remains a high priority in understanding the status of the coral reef ecosystem (Chi et al. 2021).

Environmental DNA (eDNA) is derived from metabolic waste, damaged tissue or sloughed skin cells of the organisms released into the environment and is used for detecting specific fish species through amplifying a target molecular marker or detecting multiple species through DNA metabarcoding (Kelly et al. 2014). Previous studies of DNA barcoding revealed the importance of research on large-scale fish diversity when assessing tropical biodiversity (Hubert et al. 2012). Recently, eDNA has been used to research biodiversity patterns of coral reef fishes (Gelis et al. 2021, Mathon et al. 2022, Huang et al. 2022). Compared to traditional methods, such as fishing net or diving survey, eDNA is non-invasive, efficient and reliable for investigations of marine biodiversity (Sales et al. 2020). Hainan Island harbours abundant coral-reef resources (Roder et al. 2012). Wu et al. (2015) investigated the distribution and diversity of coral reef fishes in the eastern and southern coasts of Hainan Island. However, there is no study on coral reef fish species diversity surrounding our target area, West Island, Sanya City. This paper provides some useful data to enrich the databases of Chinese tropical marine fishes, as well as possessing greatly important ecological and social significances for the conservation of the coral reef ecosystem of the South China Sea.

## Sampling methods

### Study extent

The surrounding sea area of West Island is located in the nearshore of Sanya City, Hainan Province, China.

### Sampling description

**Figure 1.**
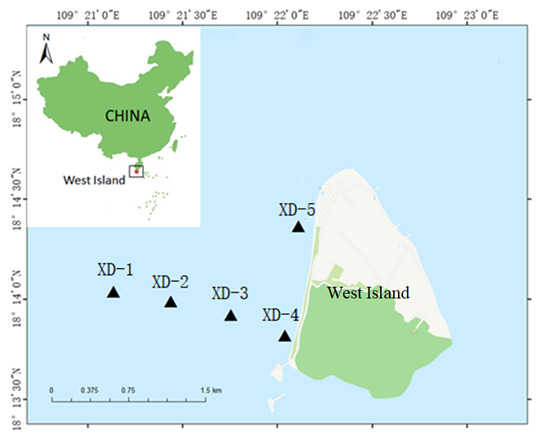
Location of eDNA sampling sites.

**Figure 2.**
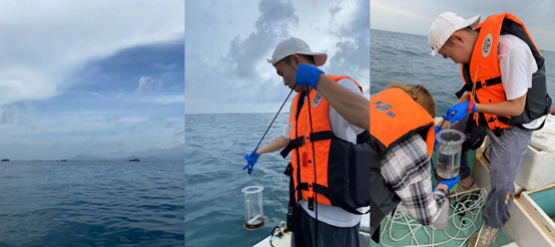
Photos of the habitat of sea area surrounding West Island and fieldwork.

The surface water samples (under water 8 m) were collected using 3 l water sampler at five localities in the sea area surrounding West Island, Hainan Province, China in June 2021 (Fig. 1). The distance between the two localities is not less than 1 km. Three replicated 2 l water samples were collected at each locality (Fig. 2). The eDNA collection was performed using the filtration method with 0.45 µm MCE membranes (Pall Whatman, U.K.). Each filtration membrane was separately preserved in a 2 ml centrifuge tube and stored at -10°C until taken back to the laboratory. The negative control was made through filtering 300 ml of pure water. Sterile masks and latex gloves were always worn during sampling and changed between different samples to prevent cross-contamination. Both the sampling devices and the filtration tools were cleaned with 10% bleach containing sodium hypochlorite to prevent external DNA contamination.

### Step description

The eDNA was extracted using the DNeasy Blood and Tissue Kit (QIAGEN, Germany) and the following modifications were made compared to the spin column protocol: a) add 720 µl ATL Buffer and 80 µl Proteinase K into centrifuge tubes containing filtration membranes and incubated 3-4 hours at 56°C; b) add 800 µl AL Buffer and 800 µl 96% ethanol after incubation; c) elution was done in 2 × 50 μl TE Buffer for a final volume of 100 μl. For each round of extraction, an extraction blank sample was made for monitoring cross-contamination, with smaller volumes being added: 180 μl ATL Buffer, 20 μl Proteinase K, 200 μl AL Buffer and 200 μl 96% ethanol. The laboratory tables, aseptic operation tables and experimental equipment were regularly cleaned with 5% bleach and then 75% ethanol before DNA extraction. The extracted DNA samples were stored at -20°C.

PCR amplification was performed using “Tele02” primers developed by Taberlet et al. (2018)(Forward: AAACTCGTGCCAGCCACC; Reverse: GGGTATCTAATCCCAGTTTG). Both forward and reverse primers were tagged with oligos designed using OligoTag (Coissac 2012) consisting of three random nucleotides and each sample was given a specific tag (Table 1). A PCR reaction total was 25 µl volume, containing 12.5 µl of 2 × Pfu PCR MasterMix (TIANGEN, China), 0.5 µl of each primer (10 µM), 0.5 µl BSA (10 mg/ml), 1µl DMSO, 0.5 µl DNaseI (5 U/μl), 4 µl of extracted DNA (10 ng/µl) and 6 µl ddH_2_O. The samples were incubated with the DNaseI for 15 min at 37°C and the DNaseI was then inactivated by incubation at 60°C for 15 min before the template DNA and the primers were added. The PCR reaction conditions were the following: pre-denaturation for 10 min at 95℃, followed by 49 cycles of denaturation (20 sec at 95°C), annealing (15 sec at 60°C) and elongation (15 sec at 72°C) and final elongation for 5 min at 72°C. PCR reactions of each sample were replicated four times. Additionally, two negative controls (PCR blank) and two positive controls (template DNA extracted from tissue of fish species *Sinocyclocheilusanophthalmus*) were in each PCR reaction, so as to monitor contamination and detect the accuracy of the target fragments, respectively. The PCR products were verified on 2% agarose gels stained with Super GelRed^TM^. In addition, the PCR products were pooled in equal volumes and each PCR pool contained one replicate of every sample. PCR blank products were also pooled together as negative controls. The PCR pools were purified using a PCR Purification Kit (QIAGEN, Germany). The libraries were built and then sequenced on an Illumina Novaseq platform (Beijing Genomics Institution, China) using 150 bp paired-end sequencing.

The raw sequences obtained from the Illumina Novaseq platform were initially processed using QIIME 2 software (Bolyen et al. 2019). The paired-end sequences were demultiplexed, based on the tags and primers were removed from the ends. Then the reads were trimmed to 160-180 bp used CUTADAPT software (Martin 2011). Next, the data obtained by sequencing was analysed by splitting, splicing and filtering to obtain high-quality sequences that were clustered into molecular operational taxonomic units (MOTUs) using VSEARCH software with ≥ 97% similarity (Zhang et al. 2019). Finally, the sequences of MOTUs were aligned with NCBI-BLAST (https://www.ncbi.nlm.nih.gov/BLAST) under the default parameters settings for taxonomic annotation in October 2021 (Djurhuus et al. 2017). We used the following criteria for taxonomic assignments: a) if the query sequence matched one single locally-occurring species in the database with ≥ 99% identity, this species was assigned; b) if the query sequence matched more than one locally-occurring species in the database with ≥ 99% identity, the lowest taxonomic level (i.e. genus or family) that included all of these species was assigned; c) if the query sequence matched one non-local species in the database with ≥ 99% identity and this species belongs to the same genus with the known local species, this genus was assigned. The sequences with an assigned taxon of ‘NA’ or assigned to human, bird, mammal or amphibian were removed. The geographic distribution of each species was evaluated by searching the FishBase website (http://www.fishbase.org/search.php).

## Geographic coverage

### Description

We surveyed five localities in the surrounding sea area of West Island (Fig. 1). The investigation involved nearly 1777 m² measured using ArcGIS 10.1 software.

### Coordinates

 and 18.230 and 18.239 Latitude Latitude; and 109.347 and 109.369 Longitude Longitude.

## Taxonomic coverage

### Description

In total, three orders, 16 families, 28 genera and 41 coral reef fish species were detected by using eDNA in the surrounding sea area of West Island.

### Taxa included

**Table:** 

Rank	Scientific Name	
kingdom	Animalia	
phylum	Chordata	
class	Osteichthyes	
order	Perciformes	
order	Clupeiformes	
order	Atheriniformes	
family	Carangidae	
family	Lutjanidae	
family	Pomacentridae	
family	Blenniidae	
family	Tripterygiidae	
family	Gerreidae	
family	Gobiidae	
family	Apogonidae	
family	Labridae	
family	Siganidae	
family	Sphyraenidae	
family	Rachycentridae	
family	Leiognathidae	
family	Clupeidae	
family	Engraulidae	
family	Atherinidae	
genus	*Trachurus*	
genus	*Trachinotus*	
genus	*Selar*	
genus	*Megalaspis*	
genus	*Alepes*	
genus	*Decapterus*	
genus	*Lutjanus*	
genus	*Abudefduf*	
genus	*Neopomacentrus*	
genus	*Blenniella*	
genus	*Enneapterygius*	
genus	*Gerres*	
genus	*Asterropteryx*	
genus	*Ostorhinchus*	
genus	*Stethojulis*	
genus	*Siganus*	
genus	*Sphyraena*	
genus	*Rachycentron*	
genus	*Hilsa*	
genus	*Herklotsichthys*	
genus	*Sardinella*	
genus	*Engraulis*	
genus	*Encrasicholina*	
genus	*Atherinomorus*	
species	*Trachurusjaponicus* (Temminck & Schlegel, 1844)	
species	*Trachinotusovatus* (Linnaeus,1758)	
species	*Trachinotusblochii* (Lacepède, 1801)	
species	*Selarcrumenophthalmus* (Bloch,1793)	
species	*Megalaspiscordyla* (Linnaeus,1758)	
species	*Alepeskleinii* (Bloch,1793)	
species	*Alepesdjedaba* (Forsskål, 1775)	
species	*Alepesmelanoptera* (Swainson,1839)	
species	*Decapterusmaruadsi* (Temminck & Schlegel,1843)	
species	Lutjanus argentimaculatus (Forsskål, 1775)	
species	*Lutjanusfulviflamma* (Forsskål, 1775)	
species	*Lutjanussebae* (Cuvier,1816)	
species	*Abudefdufvaigiensis* (Quoy & Gaimard,1825)	
species	*Abudefdufsexfasciatus* (Lacepède, 1801)	
species	*Neopomacentrusazysron* (Bleeker,1877)	
species	*Blenniellabilitonensis* (Bleeker,1858)	
species	*Enneapterygiusphilippinus* (Peters,1868)	
species	*Gerresfilamentosus* Cuvier, 1829	
species	*Asterropteryxsemipunctata* Rüppell, 1830	
species	*Ostorhinchusaureus* (Lacepède, 1802)	
species	*Stethojulisterina* Jordan & Snyder 1902	
species	*Siganusfuscescens* (Houttuyn,1782)	
species	*Sphyraenajello* Cuvier 1829	
species	*Rachycentroncanadum* (Linnaeus,1766)	
species	*Hilsakelee* (Cuvier,1829)	
species	*Herklotsichthysquadrimaculatus* (Rüppell, 1837)	
species	*Sardinellagibbosa* (Bleeker, 1849)	
species	*Engraulisjaponicus* Temminck & Schlegel, 1846	
species	*Encrasicholinapunctifer* Fowler 1938	
species	*Atherinomoruslacunosus* (Forster,1801)	
species	*Atherinomoruspinguis* (Lacepède, 1803)	
species	*Lutjanus* sp.	
species	*Abudefduf* sp.	
species	*Siganus* sp.	
species	*Sardinella* sp.1	
species	*Sardinella* sp.2	
species	*Encrasicholina* sp.	
species	Blenniidae gen. sp.	
species	Leiognathidae gen. sp.	
species	Engraulidae gen. sp.	
species	Atherinidae gen. sp.	

## Temporal coverage

### Notes

2021-6-24

## Usage licence

### Usage licence

Creative Commons Public Domain Waiver (CC-Zero)

## Data resources

### Data package title

Fish taxon-occurrences surrounding West Island, Hainan Province, China, based on eDNA

### Number of data sets

3

### Data set 1.

#### Data set name

Fish taxon-occurrences detected by eDNA

#### Data format

Darwin Core

#### Download URL

https://ipt.pensoft.net/resource?r=x-d

#### Description

The dataset presents the results of species composition of 31 coral reef fish detected in five sampling localities in the sea area surrounding West Island using eDNA techniques. The important information including taxonomic, geographic location of the occurrence and habitat condition were provided for 10 species (Xi et al. 2022 and Suppl. material 1).

**Data set 1.** 

Column label	Column description
occurrenceID	Unique occurrence identifier.
basisOfRecord	The specific nature of the data record.
eventDate	Date of sampling event.
scientificName	The full scientific name.
verbatimIdentification	A string representing the taxonomic identification as it appeared in the original record.
kingdom	The full scientific name of the kingdom in which the taxon is classified.
Phylum	The full scientific name of the phylum or division in which the taxon is classified.
Class	The full scientific name of the class in which the taxon is classified.
Order	The full scientific name of the order in which the taxon is classified.
Family	The full scientific name of the family in which the taxon is classified.
Genus	The full scientific name of the genus in which the taxon is classified.
taxonRank	The taxonomic rank of the most specific name in the scientificName as it appears in the original record.
locality	The specific description of the county from where specimens are collected.
county	The full, unabbreviated name of the next smaller administrative region than stateProvince (county, shire, department etc.) in which the Location occurs.
stateProvince	The name of the next smallest administrative region than country (state, province, canton, department, region etc.) in which the Location occurs.
Country	The full, unabbreviated name of the country where the organism was collected.
waterBody	The name of the water body in which the Location occurs.
habitat	A category or description of the habitat in which the Event occurred.
locationID	A spatial region or named place. The locationID refers to serial number of each sampling site in this study.
decimalLatitude	The geographic latitude (in decimal degrees, using the spatial reference system given in geodeticDatum) of the geographic centre of a Location.
decimalLongitude	The geographic longitude (in decimal degrees, using the spatial reference system given in geodeticDatum) of the geographic centre of a Location.
geodeticDatum	The geographic information system (GIS) upon which the geographic coordinates given in decimalLatitude, decimalLongitude and meterElevation are based.

### Data set 2.

#### Data set name

MOTUs information of coral reef fish detected by eDNA

#### Data format

Darwin Core

#### Download URL

https://www.ncbi.nlm.nih.gov/sra/PRJNA883579

#### Description

The dataset presents the nucleotides sequence and abundance of sequences of each fish species MOTU, through high-throughput sequencing of eDNA samples collected in the sea area surrounding West Island. Considering another 10 MOTUs could not be identified to definite species, and there is no single MOTU sequence, these MOTUs have not been provided in this dataset (Suppl. material 2).

**Data set 2.** 

Column label	Column description
scientificName	The full scientific name.
associatedSequences	A list (concatenated and separated) of identifiers (publication, global unique identifier, URI) of genetic sequence information associated with the Occurrence. The associatedSequences refers to MOTU sequences of each scientificName.
organismQuantity	A number or enumeration value for the quantity of organisms.
organismQuantityType	The type of quantification system used for the quantity of organisms.
dateIdentified	The date on which the subject was determined as representing the Taxon.
identificationReferences	A list (concatenated and separated) of references (publication, global unique identifier, URI) used in the Identification.
identificationRemarks	Comments or notes about the Identification.

### Data set 3.

#### Data set name

Information of environmental indicators surrounding West Island

#### Data format

Darwin Core

#### Description

The dataset presents records of seven regular marine environmental indicators, including transparency, temperature, water pressure, dissolved oxygen, electrical conductivity, hydrogen and redox potential. These records were detected at the same time when eDNA was collected (Suppl. material 3).

**Data set 3.** 

Column label	Column description
eventDate	Date of sampling event.
locationID	A spatial region or named place. The locationID refers to serial number of each sampling site in this study.
decimalLatitude	The geographic latitude (in decimal degrees, using the spatial reference system given in geodeticDatum) of the geographic centre of a Location.
decimalLongitude	The geographic longitude (in decimal degrees, using the spatial reference system given in geodeticDatum) of the geographic centre of a Location.
measurementID	An identifier for the MeasurementOrFact (information pertaining to measurements, facts, characteristics or assertions). May be a global unique identifier or an identifier specific to the dataset. The measurementID refers to serial number of each environmental sample in this study.
measurementType	The nature of the measurement, fact, characteristic or assertion. Recommended best practice is to use a controlled vocabulary.
measurementValue	The value of the measurement, fact, characteristic or assertion.
measurementUnit	The units associated with the measurementValue.
measurementDeterminedDate	The date on which the MeasurementOrFact was made.

## Additional information

**Figure 3.**
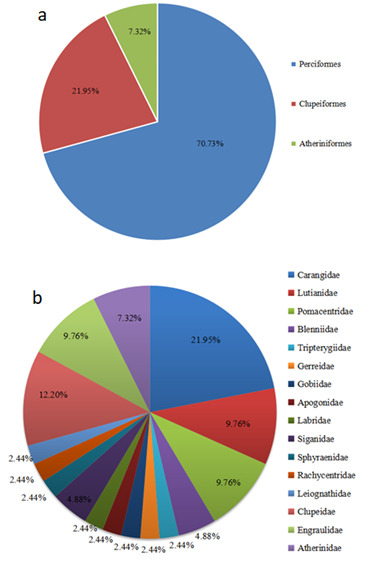
The composition of fish species of area surrounding West Island (a: at the order level; b: at the family level).

We analysed the fish composition, based on eDNA results (Fig. 3). At the order level, the order Perciformes was the most dominant and included 29 species, accounting for 70.73% of the total fish species detected; this was followed by the order Clupeiformes including nine species, accounting for 21.95% of the total number; and order Atheriniformes had the least species, including only three species and accounting for 7.32% of the total number (Fig. 3a). At the family level, the most dominant was family Carangidae and it included nine species, accounting for 21.95% of the total number; followed by family Clupeidae harbouring five species, accounting for 12.20% of total number. Next, the families Lutianidae, Pomacentridae and Engraulidae all had four species each, accounting for 9.76% of the total number, respectively; family Atherinidae included three species, accounting for 7.32% of the total number; families Blenniidae and Siganidae both included two species each, accounting for 4.88% of total number, separately; the remaining families were Tripterygiidae, Gerreidae, Gobiidae, Apogonidae, Labridae, Sphyraenidae, Rachycentridae and Leiognathidae, all including one species each, accounting for 2.44% of total number, respectively (Fig. 3b).

Herein, 31 coral reef fish species have been identified according to sequences of MOTUs amongst all species obtained. In contrast, another 10 MOTUs could not be identified to definite species, so we only represented them as indeterminate species of known genus or family, using “sp.” as the species name of them. The reason for this dilemma mainly is incomplete reference DNA database of Chinese marine fishes, especially tropical fishes. Although NCBI was still considered as a widely accepted database for alignment of DNA sequences and species annotation, data of tropical fishes are lacking (Marques et al. 2020). Therefore, it is necessary to build a reference DNA database for identifying Chinese marine fishes, especially the coral reef fishes using molecular sequences. Indeed, the good thing is that the data of our study will be helpful in building this database and improving applications of the eDNA method on the monitoring of coral reef fishes in the South China Sea. Sanya City, known as the Hawaii of China, attracts millions of travellers every year with its miles of coastline, tropical climate and some near-shore islands including West Island. Apparently, the coral reef fishes are threatened by frequent visits of these tourists. Therefore, understanding the coral reef fish composition of West Island is the very first step to prepare a sound conservation strategy. eDNA technology can also be used as an assisting tool for long-term monitoring and conservation of coral reef fishes in the surrounding islands of Sanya City.

## Supplementary Material

0FB3CCC6-561C-523D-A81A-91B42EEF4803 10.3897/BDJ.10.e89685.suppl1 Supplementary material 1 Collected fish taxon-occurrences of surrounding sea area of West Island Data type dataset Brief description This dataset provides taxonomic, distribution, habitat condition, latitude and longitude of each species of 41 coral reef-fish species detected, based on eDNA from the surrounding sea area of West Island. File: oo_733195.csv https://binary.pensoft.net/file/733195 Rui Xi, Wanru Gao, Xin Wang, Yingchun Xing

C69868BE-B9DC-56F6-8F6B-5E5FC1E304AB 10.3897/BDJ.10.e89685.suppl2 Supplementary material 2 MOTUs information of coral reef fish detected by eDNA Data type dataset Brief description This dataset provides MOTUs sequences and abundances of sequences of 31 coral-reef fish species detected by eDNA in the surrounding sea area of West Island. File: oo_747680.csv https://binary.pensoft.net/file/747680 Rui Xi, Wanru Gao, Xin Wang, Yingchun Xing

56F61948-AB6E-5E0C-8FC2-650B8EC2F126 10.3897/BDJ.10.e89685.suppl3 Supplementary material 3 Information of environmental indicators of the surrounding area of West Island Data type dataset Brief description This dataset provides the measurement values of environmental indicators of five sampling sites surrounding West Island. File: oo_707993.csv https://binary.pensoft.net/file/707993 Rui Xi, Wanru Gao, Xin Wang, Yingchun Xing

## Figures and Tables

**Table 1.** The oligo tag sequence corresponding to the samples.

Sample ID	OligoTags
XD-1	GAACTA
XD-2	AGTGTT
XD-3	AAGACA
XD-4	TACTTC
XD-5	GTCTTA
